# Barriers and Facilitators to Cervical Cancer Screening in Northern Uganda: Qualitative Insights from Healthcare Workers and Administrators

**DOI:** 10.3390/curroncol32110591

**Published:** 2025-10-23

**Authors:** Noemi Maria Felisi, David Oyet, Kayeny Miriam Melody Yung, Emmanuel Ochola, Riccardo Vecchio, Anna Odone

**Affiliations:** 1 Department of Medicine and Surgery, Università Vita-Salute San Raffaele, 20121 Milan, Italy; 2 Faculty of Public Health and Policy, London School of Hygiene and Tropical Medicine, WC1E 7HT London, UK; 3 Department of Paediatric Oncology, St. Mary’s Hospital, Lacor, Gulu P.O. Box 180, Uganda; oyet.david@lacorhospital.org; 4 Department of HIV, Research and Documentation, St. Mary’s Hospital, Lacor, Gulu P.O. Box 180, Uganda; kayenymelody@gmail.com (K.M.M.Y.); ochola.emmanuel@lacorhospital.org (E.O.); 5 Department of Public Health, Faculty of Medicine, Gulu P.O. Box 166, Uganda; 6 PhD National Programme in One Health Approaches to Infectious Diseases and Life Science Research, Department of Public Health, Experimental and Forensic Medicine, University of Pavia, 27100 Pavia, Italy; riccardo.vecchio03@universitadipavia.it; 7 Department of Public Health, Experimental and Forensic Medicine, School of Public Health, University of Pavia, 27100 Pavia, Italy; anna.odone@unipv.it; 8 Medical Direction, Fondazione IRCCS Policlinico San Matteo, 27100 Pavia, Italy

**Keywords:** cervical cancer, screening, HPV self-sampling, healthcare workers, implementation, qualitative study

## Abstract

**Simple Summary:**

Cervical cancer is the leading cause of cancer-related deaths among women in Uganda, yet screening uptake remains low. To understand how to improve access, we interviewed healthcare workers and administrators at St. Mary’s Hospital Lacor and its peripheral health centres. Participants described multi-level barriers, including limited awareness, stigma, transport costs, staff shortages, and stock-out of supplies. They also identified facilitators, such as targeted health education, routine referrals from all care entry points, outreach screening, and free services. Most participants supported self-collected HPV testing, which they felt was more acceptable to women than pelvic examination and feasible for outreach, provided supplies and laboratory capacity were sustained. These findings suggest practical strategies that facilities and policymakers can adopt to increase screening coverage. By improving awareness, referral practices, and supply reliability, and by expanding access to self-collected HPV testing, Uganda could substantially reduce the burden of cervical cancer.

**Abstract:**

Background: Cervical cancer (CC) is the most common cancer among Ugandan women and the leading cause of cancer mortality. Screening has proven to be a cost-effective method in reducing its burden, yet uptake among women of reproductive age remains alarmingly low, with national adherence rates under 10%. Objective: This study explored healthcare workers’ (HWs) perspectives on barriers and facilitators to screening and attitudes toward implementing human papillomavirus (HPV) DNA testing with self-collection. Methods: A qualitative research design was employed. Twenty semi-structured interviews were conducted with purposively sampled healthcare providers and administrators across different cadres at a referral hospital and three peripheral health centres in Northern Uganda. Interviews were analysed thematically using the Social Ecological Model. Data collection and analysis proceeded iteratively until thematic saturation. Reporting follows the Consolidated Criteria for Reporting Qualitative Research (COREQ). Results: Participants described individual and interpersonal barriers such as limited awareness, poor preventive health-seeking, fear of results, stigma, and limited male involvement. Organisational barriers included staff shortages, weak referral practices, and stock-outs of supplies, while policy constraints included limited governmental support and competing priorities. Facilitators included targeted health education, routine referrals from all service entry points, outreach screening, and donor support. Most respondents favoured scaling up of self-collected HPV testing, citing higher acceptability and feasibility for outreach, contingent on sustained supplies, laboratory capacity, and training. Conclusions: Multi-level interventions are needed to strengthen facility workflows, staff capability, community engagement, and reliable supply chains. Expanding access to self-collected HPV testing may overcome major barriers and represents a promising strategy to increase screening uptake in Uganda and similar low resource settings.

## 1. Introduction

Globally, cervical cancer (CC) ranks fourth for incidence among women, with an estimated 660,000 new cases and 350,000 deaths in 2022: approximately 90% occur in Low- and Middle-Income Countries (LMICs) [1]. Incidence has risen in sub-Saharan Africa over the past decade. In Uganda, CC is the most common female cancer and the leading cause of cancer deaths, with a crude incidence of 30 per 100,000 women and a mortality-to-incidence ratio of 0.66 [2].

In 2020, the World Health Organization (WHO) launched a global strategy to eliminate CC setting the 90-70-90 targets for 2030, aiming to fully vaccinate 90% of girls by age 15, screen 70% of women using a high-performance test by age 35 and again by age 45, and treat 90% of women with precancerous lesions or invasive cancer [3]. Uganda’s National Cervical Cancer Prevention and Control Strategic Plan (2018–2023) reflects these global objectives [4], but policy implementation gaps and health-system constraints have limited progress in prevention and control efforts [5]. 

In LMICs, logistical challenges significantly hamper the establishment of effective screening programmes, leading to low screening rates. Consequently, over 80% of women are diagnosed with advanced-stages cancer, when the five-year survival rate is below 20% [6]. Contributing factors include low awareness, insufficient or poor-quality screening programmes, limited access to services, and weak referral systems [7,8,9,10,11,12,13,14,15,16].

In Uganda, as of 2019, only 8% of eligible women had been screened in the previous five years, and merely 10% had ever been screened [17]. Visual inspection with acetic acid (VIA) was introduced in 2007 as the primary method [17]. HPV DNA testing was then introduced primarily for women living with HIV (WLHIV) given increased risk of CC [4]. It offers higher sensitivity, longer screening intervals, and strong evidence for self-collected sampling to increase participation and programmatic efficiency [18,19,20,21]. Policy mapping in East Africa underscores the need for robust national programmes and supply chains to sustain scale-up [22].

Against this background, understanding the perspectives of healthcare workers (HWs) is essential to strengthen screening delivery. This study explored HWs’ and hospital administrators’ views at a large referral hospital in Northern Uganda and its peripheral health centres, focusing on barriers and facilitators to screening, and attitudes toward expanding HPV DNA testing with self-collection. 

## 2. Materials and Methods

### 2.1. Study Design 

A qualitative, cross-sectional study was conducted to elicit HWs and administrators’ perspectives and deepen understanding of screening implementation determinants [23,24]. Health workers’ rather than women’s perspective was explored in this study due to the paucity of the former in the literature, yet provider insights are essential to provide actionable recommendations to implement the screening service. The Social Ecological Model (SEM) informed questionnaire development and analytic mapping: this model suggests that individual characteristics (such as awareness and attitude), interpersonal processes (referring to a person’s family and related network), community factors (including cultural values, beliefs, and norms), organisational factors (related to the healthcare system), and public policy factors (local and national regulations and funding) can impact the effectiveness of community prevention policies [25,26]. Reporting follows the COREQ 32-item checklist for interviews (Supplementary File S1) [27]. 

### 2.2. Study Setting 

St. Mary’s Hospital Lacor is a large Private-Not-For-Profit (PNFP) referral hospital in Gulu City, with three peripheral health centres in Opit, Amuru, and Pabbo. As the largest healthcare facility in Northern Uganda, the hospital serves a broad population, with a total capacity of 554 beds. It provides free VIA screening within the Obstetrics and Gynaecology department, and HPV DNA testing for WLHIV at the antiretroviral therapy (ART) clinic. The main hospital has a dedicated clinic room for CC screening, while peripheral centres conduct screenings within labour rooms.

### 2.3. Sampling and Recruitment

Eligibility for participation was restricted to HWs and hospital administrators with recent (past three months) work experience in CC screening or related services at the main hospital or peripheral centres. A purposive sampling method was employed to capture variation across cadre and site [28,29]. The recruitment target was to enlist 17 care providers, as previous research indicates that 9 to 17 in depth interviews can reach saturation [28]. 

A departmental staff list was provided by a hospital director; eligible participants were approached in person or by phone by the lead author (N.M.F.) to schedule an interview at a place, time and date convenient for them. Twenty participants were enrolled overall, comprising 16 HWs (clinical and medical officers, gynaecologists, laboratory technicians, midwives, nurses, palliative care specialists) and four senior administrators. While HWs could provide day-to-day clinical and operational insights, administrators were purposively included to capture organisational and policy-level determinants of service delivery, such as supply chain management, staff supervision, and resource allocation for screening activities. 

### 2.4. Data Collection 

Data were collected from October to December 2023 through semi-structured interviews. Eighteen interviews were conducted face-to-face in private rooms at the main hospital, outside working hours to maximise convenience and privacy; two were conducted via Zoom to accommodate participants at peripheral health centres. The topic guide, mapped to SEM domains, was developed to elicit participants’ perceptions and practices regarding CC screening, including barriers, facilitators and implementation requirements. It was peer-reviewed by a clinical officer with over 35 years of cancer care experience and pilot-tested with two clinical officers at Gulu University Hospital before finalisation. 

Interviews were conducted by an Italian medical doctor embedded at Lacor for several months (N.M.F.), trained by two senior researchers (A.O. and E.O.), together with a local intern (D.O.) or nurse (K.M.M.Y.). All of them spent a period working in the Obstetrics and Gynaecology department, collaborating with most of the study participants, which helped establishing rapport. Combining an outsider perspective with insider cultural competence supported confidentiality and culturally sensitive communication while preserving analytic distance. This approach is consistent with guidance on interviewer–interviewee differences and investigator triangulation [30,31]. All interviewers were fluent in English, which is widely used in Ugandan healthcare settings; therefore, no language barriers were encountered. Furthermore, they were all at the beginning of their careers, which may have helped participants feel more comfortable and less intimidated. 

The aim of the study was explained before each interview and written informed consent was obtained; confidentiality and anonymity were assured. To minimise interviewer influence and social desirability bias, interviewers used single, open questions, with neutral, non-judgemental prompts, employed active listening, and avoided leading or multiple questions. This stance facilitated rapport to generate rich, participant-led accounts [31,32].

### 2.5. Semi-Structured Interviews

A predetermined set of questions (Appendix A) was supplemented with open-ended probes to explore participants’ experiences and perspectives in relation to their professional roles, while ensuring coverage of core topics [32]. The guide comprised a brief sociodemographic section followed by four modules: (1) Knowledge and awareness of CC burden, screening methods, guidelines, and personal experiences; (2) barriers and facilitators to CC screening, including infrastructures, supplies, referral practices, challenges faced in providing routine screening, outreach, and roles of staff and administrators in supporting implementation; (3) screening methods, covering VIA experiences, views on HPV DNA testing, and implementation requirements; and (4) additional comments and recommendations. Each interview lasted approximately 45 min and was audio-recorded with permission. After the interview, the interviewers wrote brief field notes on contextual cues, including mood, gestures, and noteworthy non-verbal elements, to support interpretation and reflexivity. Participants received refreshment as a token of appreciation for participating in the study. 

### 2.6. Data Analysis

Transcripts were anonymised and analysed using the Framework Method, proceeding through the five core processes of familiarisation, development of a thematic framework, indexing (coding), charting to a case-by-code matrix, and mapping/interpretation [33,34]. An initial draft framework was developed from early transcripts and study aims and was iteratively refined during coding.

Two primary analysts (N.M.F., D.O.) manually coded all transcripts in a structured spreadsheet. A third researcher (K.M.M.Y.) independently double-coded five of the twenty transcripts to assess consistency; discrepancies were resolved by discussion to consensus, and the codebook was updated. We maintained an audit trail with versioned codebook, coding decisions, analytic memos. Coded data were charted into a framework matrix to facilitate comparison across participants. Related codes were grouped into categories and developed into themes, which were mapped to the SEM levels (individual, interpersonal, community, organisational, policy). During indexing, each code was additionally identified as a facilitator or a barrier within its SEM level [25]. Simple counts were used to indicate salience, and deviant cases were sought to challenge and refine interpretations. 

To control for data saturation, preliminary analysis proceeded alongside data collection. After coding and reviewing the 18th transcript, no new insights emerged, and existing themes were thoroughly explored. The final two transcripts confirmed consistency with the existing themes, indicating thematic saturation. 

### 2.7. Ethical Considerations 

This study was approved by the Research Ethics Committee of Lacor Hospital on 2 November 2023 with the number LACOR-2023-283. The purpose of the study and voluntary participation were explained to the participants and written consent to be involved in the study was obtained from all of them. Confidentiality was assured by using a code number instead of names and removing professional cadres to avoid identification. 

## 3. Results

### 3.1. Sample Characteristics

The sample comprised twenty participants (16 staff, 4 senior administrators). Overall, majority were males (60%), with half aged between 25 and 45 years. All of them had at least two years of practice, with half reporting over 15 years. By cadre, clinical officers were the largest group (25%), followed by medical officers (15%), midwives, gynaecologists and laboratory technicians (10% each). Administrators accounted for 20% of the sample. The sociodemographic characteristics are summarised in Table 1. 

### 3.2. Qualitative Findings

We first report participants’ knowledge and awareness of screening, then present barriers and facilitators organised by SEM level. Facilitators encompassed factors that participants had either encountered in the past or proposed as potential solutions for the future. A further paragraph is dedicated to summarize HWs’ views on scaling up HPV DNA testing. 

#### 3.2.1. Knowledge and Awareness of Cervical Cancer Screening Among Participants

Almost all interviewees recognised the high CC burden in Ugandan women. Most had working knowledge of VIA and its screen-and-treat workflow, while awareness of HPV testing was uneven: some participants had not heard of it or felt unable to compare it to VIA. Conversely, laboratory staff were confident with GeneXpert processing but less so with VIA. Knowledge of eligibility age-ranges and rescreening intervals varied: some staff referenced the 25–49 years target and 3-year VIA interval and noted annual screening for WLHIV, while others offered inconsistent ages or intervals and expressed uncertainty about updated recommendations. Across cadres, most linked their knowledge primarily to brief trainings or on-the-job learning; several requested more structured, refreshable training.

#### 3.2.2. Barriers and Facilitators to Screening Uptake by SEM Level

Main findings with corresponding exemplificative quotations are reported in Table 2. 

##### Individual Level

- Barriers

Nearly all Ps identified limited awareness and knowledge among women as the primary driver of low uptake. Lack of knowledge concerns both the significant burden of CC and the availability of free screening at Lacor Hospital: “*From my experience, cancer is very alarming, but I think it is because of lack of knowledge and awareness among our community*” (P3).

Many participants observed that preventive health-seeking was generally poor: women tended to present only when symptomatic or seriously ill, which meant that asymptomatic conditions like CC were not prioritised: “*It’s very difficult to convince women that don’t feel any pain to come for screening. They have a lot of business to run […] Cervical cancer does not pain, so they don’t come for check-up*” (P13). 

Another deterrent highlighted by many respondents was the fear of a positive screening result, as a cancer diagnosis was often perceived as a death sentence, causing significant psychological distress: “*When they hear about it, they think that maybe if they get you positive this it is going to be the end: they fear the result*” (P16).

Some HWs also identified embarrassment and discomfort linked to exposing intimate parts during the VIA test, especially with male or younger examiners, reflecting cultural barriers to sexual health discussion: “*According to the local culture, exposing your private parts is a taboo. Especially when they find a young health worker in the clinic it’s hard for them to expose their genitals*” (P6). 

Financial constraints related to transportation cost and distance were referred by over half participants as prohibitive factors; conversely, proximity to the hospital or attendance for other services were seen to facilitate uptake: “*Given this background, for some mothers it is very hard to access the hospital, especially those who live in the deep villages*” (P7).

- Facilitators

Some participants mentioned that perceived susceptibility or previous experiences of caring for a patient with CC encouraged some women to seek screening: “*There is a growing fear among people about how high the rates of cancers, not just cervical, but all cancers, it’s higher. And so people are more aware of it* (P8); “*Women who have had experience of patients with CC always have that fear of having this cancer and always come and do the investigation and screening* (P18).

##### Interpersonal Level

- Barriers

Low male involvement emerged as a consistent barrier. Participants explained that, within households, men often control financial and health-related decisions but generally showed little knowledge or interest in cervical cancer screening: “*Male involvement is very poor. Men do not have proper knowledge about CC, and even if they hear about it, their attitude kills it all because they will say* “*it doesn’t affect me as a man, but a woman*” (P15).

- Facilitators

Targeted sensitisation of male partners was viewed as an important facilitator. Joint counselling and involving men in health education sessions were thought to strengthen women’s decision-making and support with costs: “*Coming with the male partner and receiving health education together would help*” (P7).

##### Community Level

- Barriers

Stigma was frequently mentioned. In the local culture, this was often rooted in the association of CC with sexual activity. Some respondents described women facing rejection from husbands or families after a diagnosis, further exacerbating their psychological and social challenges: “*I have seen women shushed out of their homes for having the cancer and put in isolated living quarters because of the odour when the bleeding continues, in late stages. And so, the isolation and the stigmas are terrible*” (P8).

Misconceptions about screening procedures also discouraged uptake. Some community members believed that the speculum or acetic acid caused disease or infertility, or that the procedure involves the removal of the uterus. These misconceptions serve as cultural barriers to screening uptake: “*People around discourage them to come for the screening because it is thought that the instrument itself causes the disease […] or that you will not bear a child*” (P5).

- Facilitators

Raising awareness in the community was widely recognised as a facilitator. Participants stressed that additional efforts should be made to disseminate knowledge within the hospital, since women are already in the place where the screening can be taken: “*Improving dissemination, communication, passing the information are the main facilitators to encourage women to be tested*” (P2); “*Intensifying health education in the hospital is actually number one priority for making people aware, both health workers and the attendants and the patients*” (P15).

Participants stressed the importance of routine hospital health talks, outreach education, and particularly radio programmes, which reach rural audiences effectively. Encouraging women to peer share their screening experiences and involving religious and local leaders, were also suggested to raise awareness and promote screening uptake: “*When you talk on a radio, very many people will be able to listen to you. Many people in the villages have a radio*” (P3); “*More effort should be put into sensitizing the community in villages, marketplaces, schools, institutions*” (P5).

##### Organisational Level

- Barriers

Knowledge and training gaps among healthcare workers were widely reported, especially outside obstetrics and gynaecology. Some staff involved in screening activities lacked confidence in performing or interpreting screening tests: “*Knowledge gap also in the health workers is actually a barrier towards the uptake of CC screening, because if health workers don’t understand a lot about cervical cancer, they will not help a woman to go and screen*” (P15).

Weak referral practices from other hospital departments also reduced uptake. Some participants explained that sometimes they fail themselves to refer eligible women due to forgetfulness, high workloads, and competing priorities: “*Doctors mainly concentrate on the sickness which has brought the patients, […] and they forget to send them for screening*” (P12).

Another barrier mentioned was the inadequate staff available to perform screening. This shortage led to increased waiting times and interruptions in service when key staff were absent. Human resources shortages were reported especially in the peripheral health centres: “*Even if we sent all the women that come to the hospital every day, we would not have the capacity to screen all of them, because of lack of personnel in charge of the screening*” (P12).

In addition, some staff themselves have never undergone screening, while their own involvement could serve as a model for patients: “*Even health workers themselves don’t want to be screened, you have to force them. They are supposed to come first and then the other clients will come following them as an example*” (P3). 

The main practical challenge at the facility level was the shortage of supplies. Instances were reported, especially in peripheral health centres, where reagents for the VIA run out, while HPV brushes had been provided sporadically: “*Supplies are not there so we are trying to use HPV testing for those who are at higher risk*” (P10).

- Facilitators

Systematic referrals from all entry points were seen as a simple way to increase uptake, provided staff were consistent in explaining the benefits of screening, and related information such as the location of the screening clinic and its free provision: “*To increase the rate of screening in the hospital, clinicians in all entry points should be aware of the availability of the screening service and should refer all the women to do it*” (P6).

Privacy, cleanliness, and opening hours of the clinic were noted as strengths of Lacor Hospital’s service. The location of the clinic, conveniently linked to the ART and gynaecology outpatient clinics, was also noted as a facilitator. Some participants suggested that, ideally, the room should be larger, with separated sections for counselling, registration, and examination): “*I don’t know the standard, but in comparison with other places I’ve seen, I think the service offered in this hospital is better, it’s much, much better […] There is much privacy in Lacor, and the place is much better organised*” (P9). 

In a limited resource setting, free provision of services was mentioned as an important incentive: *“Those who are informed actually, come, if you tell them what it is and that you don’t attach cost to it, because cost is a very big burden”* (P10).

Finally, outreach screening campaigns, when organised by the hospital, were successful in reaching women who could not access the hospital. It was suggested to integrate them into existing outreach activities: “*Taking the activities there, to the community, that will save them from the transport costs that they fear*” (P15). 

##### Policy Level

- Barriers

Administrators described competing institutional priorities, with difficult trade-offs across programmes at the hospital, given constrained resources: “*I think prevention is huge, […] but it’s difficult to imagine increasing everything at the same time with the limited resources that the hospital has. […] Antenatal clinics and immunization clinics are absolutely a strong focus, and they always will be*” (P8).

Participants also highlighted weak governmental support and poor implementation of national policies at local level. Few facilities in the area offer screening, despite policies indicating that all peripheral health centres (HCs III and IV) should provide it: “*The government approves the policies but does not have any specific programme to effectively provide the screening*” (P12).

- Facilitators

Donor and partner support was essential to sustain free VIA and HPV testing for women living with HIV, notably through PEPFAR and private donations: “*The hospital was helped by some doctors who sponsored a free screening, which is encouraging many women to come*” (P9).

Participants recommended operationalising national plans by ensuring reliable supplies at all levels, running nationwide awareness campaigns, expanding screening at all health centres and implementing outreach to reduce distance and information barriers: “*If they can try as much as possible to make these supplies ready or available most of the times, I think it would be beneficial. […] Then for the policymakers, maybe they can run a programme that will remind the population about CC and its burden in the country*” (P10).

#### 3.2.3. Health Workers’ View on Scaling HPV DNA Testing

This theme synthesises perspectives on implementing HPV DNA testing, including self-collection, across cadres with direct experience of VIA or HPV DNA workflows. Views across cadres were triangulated to compare perceived advantages, constraints, and implementation requirements. Findings are presented at individual, organisational, and policy levels, no interpersonal or community themes were identified (Table 3).

##### Individual Level

Most clinicians described HPV DNA testing, especially via self-collection, as more acceptable to women, avoiding speculum examination and associated embarrassment and concerns. This was perceived to increase willingness to screen, with some women clearly expressing their preference for HPV testing: “*HPV DNA could benefit the women and attract more women, because it’s easy. They do it as a self-test, the women do it and produce the sample. Even women fear the speculum*” (P11); “*Sometimes [HIV] negative clients come to me saying that they want to do it (HPV DNA test), but I just tell them that they have to go for VIA*” (P3).

Participants also emphasized higher sensitivity of HPV DNA testing, enabling earlier identification of women at risk and longer screening intervals: “*HPV DNA screening should be the best because if someone is infected with HPV you could easily follow this at risk over some time. With VIA we don’t know who’s exposed, we are just looking for the result of the exposure*” (P9).

##### Organisational Level

Some respondents stressed that self-collection of HPV testing is feasible in community settings, whereas VIA requires a private, sterile room and equipment, rendering it impractical during outreach: “*At outreaches we cannot integrate the screening because we don’t have specific rooms where we can really maintain privacy […]. When the samples of HPV are there, it is ok because the mothers pick the samples themselves and we just bring the samples to be run in Lacor […]. HPV DNA test is easier to be done in the communities*” (P16).

Several participants perceived HPV testing as simpler and faster to deliver and interpret, thereby reducing staff workload. By contrast, VIA requires experienced and well-trained personnel to interpret results accurately: “*The DNA test is faster and does not require a lot of work, you just need to explain the women how to do it and pick the sample, while with VIA you need to sit, put the speculum before you do the test* etc.” (P13).

However, many participants noted that, unlike VIA’s immediate result and treatment, HPV testing requires at least one day for laboratory analysis. Delays in communicating results could impact patients’ outcomes, especially for outreach and peripheral health centres, as samples must return to the main facility. Limited phone access also impede recall of positive clients, undermining timely triage and treatment. “Most times the results come back when the women are not there, they have gone far, they don’t have contacts, so you have to wait until they come back for a routine appointment to give them the result” (P17). Should the use of HPV DNA test increase, there would also be a need to enhance the laboratory capacity and efficiency in processing and delivering results: “The practicability of implementing the HPV DNA test depends on the possibility to improve the waiting time before receiving the results of HPV test and if we were able to communicate the results in time to the patients by calling them” (P2).

Additionally, laboratory staff stressed the need for clear, brief training on self-collection to minimize invalid samples: “I think GeneXpert is highly sensitive but that depends on how the sample has been collected. When it is not done correctly, you end up with errors” (P19). 

Finally, a few HWs highlighted the limited familiarity with the HPV DNA test compared to the VIA, given their greater exposure to the latter. Targeted training and adaptation to the former are essential: “*I’m not so much, you know, exposed to it [HPV DNA test]. That’s why I can’t do much about it. When you train the health workers it becomes easier*” (P10). 

##### Policy Level

The main perceived barriers to scale-up were the high cost of supplies and the frequent shortage of brushes and kits. Donor dependence and the lack of governmental policies guaranteeing access to all women pose significant obstacles to its adoption beyond WLHIV: “*Right now, we are able to offer HPV test because a donor provided but then when it is over we cannot sustain it*” (P12). 

Overall, most HWs expressed favourable attitudes toward scaling up the HPV test for CC screening among WLHIV and supported extending its use to the general population of women, conditional on reliable supplies, strengthened laboratory capacity, effective recall system, and training for providers and clients. Most HWs expressed their preference for the HPV DNA test for both outreaches and facilities. 

## 4. Discussion

This qualitative study at a large referral hospital in Northern Uganda and its peripheral centres identified multi-level determinants of cervical cancer (CC) screening uptake and explored healthcare workers (HWs)’ views on scaling HPV DNA testing. Using the Social Ecological Model enabled analysis across individual, interpersonal, community, organisational, and policy domains. A secondary aim explored attitudes toward scaling HPV DNA testing with self-collection, due to its potential to increase screening rate, by overcoming some of the socioeconomic and logistical barriers experienced with the VIA [35,36,37], and in line with WHO guidance [38]

Consistent with prior studies, perceived susceptibility, often prompted by indirect experience of cancer, emerged as a motivator for screening [11,39], while limited awareness regarding CC and available screening services were repeatedly cited as barriers [40,41]. Stigma, fear of results, and misconceptions about screening procedures mirrored findings from LMIC contexts [6,7,8,9,12,13,15,16,42,43,44,45]. Distance and transport costs also constrained uptake, as reported in other rural settings [10,46,47,48]; outreach screening and expanding services to peripheral health centres were proposed to mitigate these access barriers [16,49]. In contrast, hospital-based health education and community awareness campaigns, including radio talk shows and engagement of local leaders, were emphasized as facilitators, as well as male involvement [39,50,51].

At facility level, participants reported gaps in staff knowledge and confidence, expressing the need of additional educational and training sessions [10,52]. Low HWs awareness contributed to weak referral practices from other departments, mirroring findings from LMIC settings [8,26]. Participants recommended regular in-service training to normalize referral of all eligible women and stressed the value of clear messaging about clinic location, opening hours and the free nature of services [9,53,54]. Staff shortfalls, especially in the peripheral health centres, increased waiting times and disrupted services [9,12,55,56], whereas privacy, cleanliness and convenient opening hours were perceived facilitators [14,37,46].

Administrators highlighted prevention as a priority but acknowledged difficult trade-offs under resource constraints. As in an earlier Ugandan study, participants perceived national policies as weakly operationalized locally, with few facilities offering screening, despite policy intent [57]. Donor support underpinned free VIA and HPV testing for WLHIV, but dependence on external funding and the cost of HPV kits limited scale. 

Regarding screening modality, HPV DNA testing, particularly self-collection, was perceived as more acceptable than speculum-based VIA and operationally better suited to outreach [58,59,60,61]. They highlighted its higher sensitivity and potential for longer screening intervals [18,19,20,21,62,63], and evidence of clinical benefit in LMIC contexts as well as strong performance of self-collected samples [64,65]. However, they also noted programme requirements for scale-up: training to ensure adequate self-sampling, laboratory capacity and timely result return, reliable recall systems for triage of patients turning positive, and steady supplies. Taken together, these findings support a phased expansion of HPV DNA testing beginning with outreach-compatible self-collection, coupled with workflow investments to shorten turnaround time and reduce loss-to-follow-up.

A key strength of this study lies in its implementation focus: findings translate into practical intervention strategies for facilities and policymakers in high-burden, resource-constrained settings. The use of the Social Ecological Model helped structure questioning and analysis, making explicit how barriers and facilitators from individual to policy levels influence screening uptake. A healthcare-centred perspective was adopted, focusing especially on organisational and policy-related factors, since these have been less explored in the previous literature. The inclusion of hospital administrators alongside frontline HWs strengthened the analysis by incorporating managerial perspectives on screening delivery. 

Several limitations should be noted. First, this is a single-system, cross-sectional study, which may limit generalisation beyond comparable facilities. Second, women’s perspectives were not directly elicited: individual-level factors reflect providers’ reports, which may omit salient determinants. However, women’s perspectives are well documented in the literature, and limited time and resources for this study constrained the scope to one stakeholder group. Third, the predominance of male participants, reflecting the gender composition of staff at the study sites, may have limited the understanding of women’s perception of invasive exams and screening procedures. Fourth, despite mitigation efforts, social desirability and positionality biases could have shaped responses, given respondents’ roles and responsibilities within the hospital, particularly for managerial positions. Fifth, we did not directly observe clinic workflows or link themes to service data, limiting causal inference about specific bottlenecks and the likely impact of proposed solutions. Finally, purposive sampling may have under-represented certain cadres or dissenting views; participation was voluntary and could favour more engaged staff.

## 5. Conclusions

This study explored HWs’ perspectives on factors associated with cervical cancer screening in Northern Uganda and their view on scaling HPV DNA testing. Findings highlight the critical role of HWs in increasing screening uptake, by raising awareness within facilities and communities, encouraging regular screening, fostering male involvement, and addressing fears and misconceptions. Hospital administrators can support screening uptake though organisational interventions, such as staff training, maintaining high-quality screening services, and minimizing waiting times. Lastly, policymakers should prioritize the implementation of screening programmes in rural areas and ensure reliable supply chains. Innovative techniques, such as self-administered HPV testing, were widely viewed as acceptable and feasible, with the potential to overcome VIA-related challenges. However, sustainable adoption requires reliable supplies, strengthened laboratory capacity, and integration beyond HIV-related campaigns. Overall, these insights provide actionable guidance for practitioners, administrators, and policymakers. By addressing barriers across system levels and expanding access to HPV self-collection, screening uptake can be increased, contributing to reduced CC burden in Uganda and similar settings.

## Figures and Tables

**Table 1:** Sociodemographic characteristics of participating healthcare workers and administrators (*n* = 20).

Characteristics	*n* (%)
Gender	
Male	12 (60)
Female	8 (40)
Age (Years)	
25–45	10 (50)
46–60	8 (40)
61 and above	2 (10)
Years of Practice	
2–5	4 (20)
6–15	6 (30)
>15	10 (50)
Role	
Clinical Officer	5 (25)
Medical Officer	3 (15)
Midwife	2 (10)
Gynaecologist	2 (10)
Laboratory Technician	2 (10)
Nurse	1 (5)
Palliative Care Specialist	1 (5)
Administrators	4 (20)

**Table 2:** Barriers and Facilitators of CC Screening at Lacor Hospital.

SEM Level		Barriers	Extract/Example
Individual	Barriers	Poor women awareness	“Cancer is very alarming, because of lack of knowledge and awareness among our community” (P3)
		Poor health-seeking	“It’s very difficult to convince women that don’t feel any pain to come for screening” (P13)
		Fear of results	“When they hear about it, they think that maybe if they get you positive this it is going to be the end: they fear the result.” (P16)
		Accessibility	“For some mothers it is very hard to access the hospital, especially those who live in the deep villages.” (P7)
		Procedure-related embarrassment and discomfort	“There is fear of being enlarged, because in their marriage life they don’t feel comfortable. So, they are discouraged by the community.” (P15)
	Facilitators	Fear of CC	“There is a growing fear about high rates of cancers… And so people are more aware of it.” (P8)
		Closeness to CC patients	“Women who have had experience of patients with cervical cancer always have fear of having this cancer and come to screening” (P18)
Interpersonal	Barriers	Low male involvement	“Male involvement is very poor.” (P15)
	Facilitators	Men sensitisation	“Coming with the male partner and receiving health education together would help” (P7)
Community	Barriers	Stigma	“Isolation and the stigmas are terrible.” (P8)
		Misconceptions related to VIA procedure	“People around discourage them to come for the screening because it is thought that the instrument itself causes the disease.” (P5)
	Facilitators	Raise awareness in the community	“Improving dissemination, communication, passing the information are the main facilitators to encourage women to be tested.” (P2)
Organisational	Barriers	Poor HWs knowledge	“If health workers don’t understand a lot about CC, they will not help a woman to go and screen.” (P15)
		Poor HWs attitude	“Doctors mainly concentrate on the sickness which has brought the patients,… and later on they forget to send them for screening.” (P12)
		Inadequate staff	“I think that the low personnel dedicated to screening and having the knowledge of how to do it contributes to the low screening.” (P2)
		Shortage of supplies	“Supplies are not there so we are trying to use it [HPV DNA test] for those who are at higher risk.” (P10)
	Facilitators	Reinforcing referral practices	“Clinicians in all entry points should be aware of the availability of the screening service and should refer all the women.” (P6)
		Privacy, location, cleanliness and opening hours of the screening clinic	“I think the service offered in this hospital is better… There is much privacy in Lacor, and the place is much better organised.” (P9)
		Free service	“Those who are informed, actually come, if you tell them what it is and that you don’t attach cost to it, because cost is a very big burden.” (P10)
		Outreach screenings	“Another motivator is narrowing the distance, which means to take these activities to the community where these women are and they’re not able to come to the hospital.” (P15)
Policy	Barriers	Competing administrative priorities	“It’s difficult to imagine increasing everything at the same time with the limited resources that the hospital has” (P8)
		Poor governmental support	“The government approves the policies but does not have any specific programme to effectively provide the screening.” (P12)
		Low number of screening points	“The facilities that do screening are not many… nearby health centres don’t do it.” (P15)
	Facilitators	Partners’ and donors’ support	“The hospital was helped by some doctors who sponsored a free screening, which is encouraging many women to come.” (P9)
		National implementation of local screening programmes	“…maybe they can run a programme that will remind the population about cervical cancer and its burden in the country.” (P10)

P: Participant.

**Table 3:** Comparison of VIA and HPV DNA testing based on healthcare workers’ perspectives.

SEM Level	Domain	VIA	HPV DNA Testing
Individual	Acceptability	Embarrassment, discomfort, and fear related to pelvic examination.	More women-friendly, self-collection mitigates modesty concerns.
	Clinical performance	Lower sensitivity; shorter screening interval.	Higher sensitivity; longer intervals, earlier risk identification; requires triage.
Organisational	Workload and setting fit	Needs private, sterile room and trained examiner.	Feasible in outreach; reduced workload for hospital staff.
	Result interpretation	Operator-dependent; requires experience.	Laboratory tests perceived as easier to interpret.
	Laboratory	No laboratory processing required.	Dependent on GeneXpert capacity.
	Result return and follow-up	Immediate result communication and treatment	Requires triage after positive results (VIA/ colposcopy); recall challenged by limited phone access.
	Training and familiarity	Widely familiar among staff.	Targeted training needed.
Policy	Supplies and costs	VIA reagents generally available; low cost.	Variable brush and kits availability; donor-dependent; high kit cost.

## Data Availability

The qualitative data generated and analysed during the current study are not publicly available to protect the anonymity of our participants, but de-identified data may be made available from the corresponding author on reasonable request.

## Supplementary Materials

The following supporting information can be downloaded at: https://www.mdpi.com/article/10.3390/curroncol32110591/s1, Supplementary File S1: COREQ 32-item checklist.

## Abbreviations

The following abbreviations are used in this manuscript:

ART	Antiretroviral Therapy
CC	Cervical Cancer
COREQ	Consolidated Criteria for Reporting Qualitative Research
HIV	Human Immunodeficiency Virus
HPV	Human Papillomavirus
HWs	Healthcare Workers
LMICs	Low- and Middle-Income Countries
P	Participant
PEPFAR	President’s Emergency Plan for AIDS Relief
PNFP	Private Not-for-Profit
SEM	Social Ecological Model
VIA	Visual Inspection with Acetic Acid
WHO	World Health Organization.
WLHIV	Women Living with HIV

## Appendix A

### Appendix A.1. Semi-Structured Interview Guide

#### Appendix A.1.1. Section 1： Participants’ Sociodemographic Characteristics

- Gender
- Age
- Professional cadre
- Years of practice in the health field

#### Appendix A.1.2. Section 2： Knowledge and Awareness of Cervical Cancer

1. What can you tell me about cervical cancer and its screening?
  (prompts: incidence and mortality in Uganda, WHO 2030 targets)
2. What do you know about the official guidelines for cervical cancer screening?
  (prompts: age bracket, methods, recommended intervals)
3. How did you learn about cervical cancer? What training or continuing education have you received?
  (prompts: CME, conferences, seminars, medical education, social media)
4. What methods does this health facility use for cervical cancer screening, and what do you know about these methods?
  (prompts: Pap smear, VIA, HPV DNA test)
5. What experience do you have with cervical cancer screening in this hospital?
  (prompts: provider, referral, counsellor, policymaker roles)

#### Appendix A.1.3. Section 3： Barriers and Facilitators to Screening

1. In your experience, what are the main barriers to screening uptake for women at the individual, social, or cultural level?
  (prompts: knowledge, fear, embarrassment, low perceived risk, poor valuation of screening, lack of spousal/family support, gender inequality)
2. What are the main factors that motivate women to undergo screening?
  (prompts: awareness, education, employment, external support, symptoms, health worker recommendation)
3. How would you judge the adequacy of infrastructures and resources for CC screening in this hospital?
  (prompts: space and privacy, clinic cleanliness, opening hours, waiting times, links to HIV and other departments; human resources—training, number of staff; materials for screening and treatment)
4. What challenges or barriers do you and colleagues face in providing routine screening?
  (prompts: costs, inadequate supplies, limited investment, inconvenient clinic times, high staff turnover)
5. How have you and your colleagues addressed these challenges?
6. What is the hospital doing to increase cervical cancer screening rates in the community?
  (prompts: outreach activities, peripheral health centres)
7. [For staff] What do you do in your daily work to support the implementation of screening?
  (prompts: prioritising CC screening, counselling, empowering women, sensitising patients)
8. [For administrators] What would be required to motivate staff to routinely follow guidelines?
  (prompts: incentives, training)
9. [For administrators] How does CC screening compare to other hospital programmemes in terms of importance and value?
  (prompts: resources, attention, training, investments)
10. What suggestions do you have for national and local policymakers?

#### Appendix A.1.4. Section 4： Screening Methods

1. In your opinion, what are the advantages and disadvantages of HPV DNA testing compared to VIA?
2. What is your attitude toward the implementation of HPV DNA testing in this hospital?
3. What do you see as the main barriers to its implementation here?
  (prompts: high costs, inadequate supplies, integration challenges, limited training or knowledge)
4. What changes would be required for adopting HPV DNA testing as a routine method in the screening programme?

#### Appendix A.1.5. Section 5： Closing

1. Is there anything else related to cervical cancer screening that you want to tell?

## References

[1] Bray F., Laversanne M., Sung H., Ferlay J., Siegel R.L., Soerjomataram I., Jemal A. (2024). Global cancer statistics 2022: GLOBOCAN estimates of incidence and mortality worldwide for 36 cancers in 185 countries. CA Cancer J. Clin..

[2] WHO (2021). Uganda, Cervical Cancer Profile. https://cdn.who.int/media/docs/default-source/country-profiles/cervical-cancer/cervical-cancer-uga-2021-country-profile-en.pdf.

[3] World Health Organization (2020). Global Strategy to Accelerate the Elimination of Cervical Cancer as a Public Health Problem.

[4] The Republic of Uganda Ministry of Health (2018). The National Cervical Cancer Prevention and Control Strategic Plan [Internet].

[5] Obol J.H., Lin S., Obwolo M.J., Harrison R., Richmond R. (2021). Knowledge, attitudes, and practice of cervical cancer prevention among health workers in rural health centres of Northern Uganda. BMC Cancer.

[6] Petersen Z., Jaca A., Ginindza T.G., Maseko G., Takatshana S., Ndlovu P., Zondi N., Zungu N., Varghese C., Hunting G. (2022). Barriers to uptake of cervical cancer screening services in low-and-middle-income countries: A systematic review. BMC Womens Health.

[7] Adewumi K., Nishimura H., Oketch S.Y., Adsul P., Huchko M. (2022). Barriers and Facilitators to Cervical Cancer Screening in Western Kenya: A Qualitative Study. J. Cancer Educ..

[8] Ebu N.I. (2018). Facilitators and barriers to cervical cancer screening among HIV-positive women in Ghana. Afr. J. Midwifery Womens Health.

[9] Lott B.E., Halkiyo A., Kassa D.W., Kebede T., Dedefo A., Ehiri J., Madhivanan P., Carvajal S., Soliman A. (2021). Health workers’ perspectives on barriers and facilitators to implementing a new national cervical cancer screening programme in Ethiopia. BMC Womens Health.

[10] Darj E., Chalise P., Shakya S. (2019). Barriers and facilitators to cervical cancer screening in Nepal: A qualitative study. Sex. Reprod. Healthc..

[11] Ndejjo R., Mukama T., Kiguli J., Musoke D. (2017). Knowledge, facilitators and barriers to cervical cancer screening among women in Uganda: A qualitative study. BMJ Open.

[12] Bevilacqua K.G., Gottschlich A., Murchland A.R., Alvarez C.S., Rivera-Andrade A., Meza R. (2022). Cervical cancer knowledge and barriers and facilitators to screening among women in two rural communities in Guatemala: A qualitative study. BMC Womens Health.

[13] Boni S.P., Gnahatin F., Comoé J.C., Tchounga B., Ekouevi D., Horo A., Adoubi I., Jaquet A. (2021). Barriers and facilitators in cervical cancer screening uptake in Abidjan, Côte d’Ivoire in 2018: A cross-sectional study. BMC Cancer.

[14] Vega Crespo B., Neira V.A., Ortíz Segarra J., Andrade A., Guerra G., Ortiz S., Flores A., Mora L., Verhoeven V., Gama A. (2022). Barriers and facilitators to cervical cancer screening among under-screened women in Cuenca, Ecuador: The perspectives of women and health professionals. BMC Public Health.

[15] Chua B., Ma V., Asjes C., Lim A., Mohseni M., Wee H.L. (2021). Barriers to and Facilitators of Cervical Cancer Screening among Women in Southeast Asia: A Systematic Review. Int. J. Environ. Res. Public Health.

[16] Black E., Hyslop F., Richmond R. (2019). Barriers and facilitators to uptake of cervical cancer screening among women in Uganda: A systematic review. BMC Womens Health.

[17] ICO/IARC Information Centre on HPV and Cancer (2023). Uganda: Human Papillomavirus and Related Cancers, FactSheet 2023. https://hpvcentre.net/statistics/reports/UGA_FS.pdf.

[18] Tang J.H., Lee F., Chagomerana M.B., Ghambi K., Mhango P., Msowoya L., Mkochi T., Magongwa I., Mhango E., Mbendera J. (2023). Results from Two HPV-Based Cervical Cancer Screening-Family Planning Integration Models in Malawi: A Cluster Randomized Trial. Cancers.

[19] Le Goff J., Le Duc-Banaszuk A.S., Lefeuvre C., Pivert A., Ducancelle A., De Pauw H., Arbyn M., Vinay A., Rexand-Galais F. (2023). Acceptability to Healthcare Professionals of Home-Based HPV Self-Sampling for Cervical Screening: A French Qualitative Study Conducted in an Area with Low Access to Health Services. Cancers.

[20] Sørbye S.W., Falang B.M., Botha M.H., Snyman L.C., van der Merwe H., Visser C., Richter K., Dreyer G. (2023). Enhancing Cervical Cancer Prevention in South African Women: Primary HPV mRNA Screening with Different Genotype Combinations. Cancers.

[21] Daponte N., Valasoulis G., Michail G., Magaliou I., Daponte A.I., Garas A., Garas A., Grivea I., Bogdanos D.P., Daponte A. (2023). HPV-Based Self-Sampling in Cervical Cancer Screening: An Updated Review of the Current Evidence in the Literature. Cancers.

[22] Njuguna D.W., Mahrouseh N., Onisoyonivosekume D., Varga O. (2020). National policies to prevent and manage cervical cancer in East African countries: A policy mapping analysis. Cancers.

[23] Williams V., Boylan A.M., Nunan D. (2020). Critical appraisal of qualitative research: Necessity, partialities and the issue of bias. BMJ Evid. Based Med..

[24] Neubauer B.E., Witkop C.T., Varpio L. (2019). How phenomenology can help us learn from the experiences of others. Perspect. Med. Educ..

[25] Golden S.D., Earp J.A.L. (2012). Social Ecological Approaches to Individuals and Their Contexts: Twenty Years of Health Education & Behavior Health Promotion Interventions. Health Educ. Behav..

[26] Nyambe A., Van Hal G., Kampen J.K. (2016). Screening and vaccination as determined by the Social Ecological Model and the Theory of Triadic Influence: A systematic review. BMC Public Health.

[27] Tong A., Sainsbury P., Craig J. (2007). Consolidated criteria for reporting qualitative research (COREQ): A 32-item checklist for interviews and focus groups. Int. J. Qual. Health Care.

[28] Hennink M., Kaiser B.N. (2022). Sample sizes for saturation in qualitative research: A systematic review of empirical tests. Soc. Sci. Med..

[29] Etikan I. (2016). Comparison of Convenience Sampling and Purposive Sampling. Am. J. Theor. Appl. Stat..

[30] Carter N., Bryant-Lukosius D., Dicenso A., Blythe J., Neville A.J. (2014). The use of triangulation in qualitative research. Vol. 41, Oncology Nursing Forum. Oncol. Nurs. Soc..

[31] Durand M.A., Chantler T. (2014). Principles of Social Research, 2nd ed.

[32] DeJonckheere M., Vaughn L.M. (2019). Semistructured interviewing in primary care research: A balance of relationship and rigour. Fam. Med. Community Health.

[33] Gale N.K., Heath G., Cameron E., Rashid S., Redwood S. (2013). Using the framework method for the analysis of qualitative data in multi-disciplinary health research. BMC Med. Res. Methodol..

[34] Kallio H., Pietilä A.M., Johnson M., Kangasniemi M. (2016). Systematic methodological review: Developing a framework for a qualitative semi-structured interview guide. J. Adv. Nurs..

[35] Camara H., Zhang Y., Lafferty L., Vallely A.J., Guy R., Kelly-Hanku A. (2021). Self-collection for HPV-based cervical screening: A qualitative evidence meta-synthesis. BMC Public Health.

[36] Brandt T., Wubneh S.B., Handebo S., Debalkie G., Ayanaw Y., Alemu K., Jede F., Doeberitz M.v.K., Bussmann H. (2019). Genital self-sampling for HPV-based cervical cancer screening: A qualitative study of preferences and barriers in rural Ethiopia. BMC Public Health.

[37] Nyabigambo A., Mayega R.W., Hlongwana K., Ginindza T.G. (2023). Facilitators and Barriers to HPV Self-Sampling as a Cervical Cancer Screening Option among Women Living with HIV in Rural Uganda. Int. J. Environ. Res. Public Health.

[38] World Health Organization (2021). WHO Guideline for Screening and Treatment of Cervical Pre-Cancer Lesions for Cervical Cancer Prevention.

[39] Paul P., Winkler J.L., Bartolini R.M., Penny M.E., Huong T.T., Nga L.T., Kumakech E., Mugisha E., Jeronimo J. (2013). Screen-and-Treat Approach to Cervical Cancer Prevention Using Visual Inspection with Acetic Acid and Cryotherapy: Experiences, Perceptions, and Beliefs From Demonstration Projects in Peru, Uganda, and Vietnam. Oncologist.

[40] Teame H., Gebremariam L., Kahsay T., Berhe K., Gebreheat G., Gebremariam G. (2019). Factors affecting utilization of cervical cancer screening services among women attending public hospitals in Tigray region, Ethiopia, 2018; Case control study. PLoS ONE.

[41] Shiferaw S., Addissie A., Gizaw M., Hirpa S., Ayele W., Getachew S., Kantelhardt E.J., Assefa M., Jemal A. (2018). Knowledge about cervical cancer and barriers toward cervical cancer screening among HIV-positive women attending public health centres in Addis Ababa city, Ethiopia. Cancer Med..

[42] Williams M.S., Kenu E., Adanu A., Yalley R.A., Lawoe N.K., Dotse A.S., Adu R.F., Fontaine K. (2019). Awareness and Beliefs About Cervical Cancer, the HPV Vaccine, and Cervical Cancer Screening Among Ghanaian Women with Diverse Education Levels. J. Cancer Educ..

[43] Canfell K., Kim J.J., Brisson M., Keane A., Simms K.T., Caruana M., Burger E.A., Martin D., Nguyen D.T.N., Bénard É. (2020). Mortality impact of achieving WHO cervical cancer elimination targets: A comparative modelling analysis in 78 low-income and lower-middle-income countries. Lancet.

[44] Ndejjo R., Mukama T., Musabyimana A., Musoke D. (2016). Uptake of Cervical Cancer Screening and Associated Factors among Women in Rural Uganda: A Cross Sectional Study. PLoS ONE.

[45] Mailhot Vega R.B., Balogun O.D., Ishaq O.F., Bray F., Ginsburg O., Formenti S.C. (2019). Estimating child mortality associated with maternal mortality from breast and cervical cancer. Cancer.

[46] Binka C., Nyarko S.H., Awusabo-Asare K., Doku D.T. (2019). Barriers to the Uptake of Cervical Cancer Screening and Treatment among Rural Women in Ghana. Biomed. Res. Int..

[47] Bien-Aimé D.D.R. (2020). Understanding the Barriers and Facilitators to Cervical Cancer Screening Among Women in GONAIVES, Haiti: An Explanatory Sequential Mixed-Methods Study.

[48] Daghighbin E., Najar A., Tehrani H., Saghi F., Ghavami V., Houshmand E., Ebrahimipour H. (2023). Using social marketing theory as a framework for understanding barriers and facilitators of human papillomavirus screening in women: A qualitative study. J. Educ. Health Promot..

[49] Basagoitia A., Burrowes S., Solis-Soto M.T., MacMillan G., Sullivan S. (2023). Community and provider perceptions and experiences of cervical cancer screening in Rural Bolivia: A qualitative study. BMC Womens Health.

[50] Buchanan Lunsford N., Ragan K., Lee Smith J., Saraiya M., Aketch M. (2017). Environmental and Psychosocial Barriers to and Benefits of Cervical Cancer Screening in Kenya. Oncologist.

[51] Mwaka A.D., Wabinga H.R., Mayanja-Kizza H. (2013). Mind the gaps: A qualitative study of perceptions of healthcare professionals on challenges and proposed remedies for cervical cancer help-seeking in post conflict northern Uganda. BMC Fam. Pract..

[52] Nguyen-Truong C.K.Y., Hassouneh D., Lee-Lin F., Hsiao C.Y., Le T.V., Tang J., Vu M., Truong A.M. (2018). Health Care Providers’ Perspectives on Barriers and Facilitators to Cervical Cancer Screening in Vietnamese American Women. J. Transcult. Nurs..

[53] Liebermann E.J., VanDevanter N., Shirazian T., Frías Gúzman N., Niles M., Healton C., Ompad D. (2020). Barriers to Cervical Cancer Screening and Treatment in the Dominican Republic: Perspectives of Focus Group Participants in the Santo Domingo Area. J. Transcult. Nurs..

[54] Ciceron A.C., Jeon M.J., Monroe A.K., Clausen M.E., Magnus M., Le D. (2022). HPV knowledge, screening barriers and facilitators, and sources of health information among women living with HIV: Perspectives from the DC community during the COVID-19 pandemic. BMC Womens Health.

[55] Tshabalala G., Blanchard C., Mmoledi K., Malope D., O’Neil D.S., Norris S.A., Joffe M., Dietrich J.J. (2023). A qualitative study to explore healthcare providers’ perspectives on barriers and enablers to early detection of breast and cervical cancers among women attending primary healthcare clinics in Johannesburg, South Africa. PLOS Glob. Public Health.

[56] Girardi D., Vecchio R., Tanious M., Cacitti S., Ancarani C., Dalle Carbonare S., Perotti P., Bonafede C., Cavallo R., Gentile L. (2024). Disparities in access to breast, colorectal, and cervical cancer screening programmes have intensified during the pandemic period. Findings of a health equity audit conducted by the Pavia Healthcare Protection Agency (Lombardy Region, Northern Italy). Epidemiol. Prev..

[57] Obol J.H., Harrison R., Lin S., Obwolo M.J., Richmond R. (2020). Perceptions of key informants on the provision of cervical cancer prevention and control programme in Uganda: Implication for cervical cancer policy. BMC Public Health.

[58] Xiong S., Lazovich D.A., Hassan F., Ambo N., Ghebre R., Kulasingam S., Mason S.M., Pratt R.J. (2022). Health care personnel’s perspectives on human papillomavirus (HPV) self-sampling for cervical cancer screening: A pre-implementation, qualitative study. Implement. Sci. Commun..

[59] Sultanov M., de Zeeuw J., Koot J., van der Schans J., Beltman J.J., de Fouw M., Majdan M., Rusnak M., Nazrul N., Rahman A. (2022). Investigating feasibility of 2021 WHO protocol for cervical cancer screening in underscreened populations: PREvention and SCReening Innovation Project Toward Elimination of Cervical Cancer (PRESCRIP-TEC). BMC Public Health.

[60] Mensah K., Assoumou N., Duchesne V., Pourette D., DeBeaudrap P., Dumont A. (2020). Acceptability of HPV screening among HIV-infected women attending an HIV-dedicated clinic in Abidjan, Côte d’Ivoire. BMC Womens Health.

[61] Wood B., Lofters A., Vahabi M. (2018). Strategies to Reach Marginalized Women for Cervical Cancer Screening: A Qualitative Study of Stakeholder Perspectives. Curr. Oncol..

[62] Rawat A., Sanders C., Mithani N., Amuge C., Pedersen H., Namugosa R., Payne B., Mitchell-Foster S., Orem J., Ogilvie G. (2021). Acceptability and preferences for self-collected screening for cervical cancer within health systems in rural Uganda: A mixed-methods approach. Int. J. Gynecol. Obstet..

[63] Mezei A.K., Armstrong H.L., Pedersen H.N., Campos N.G., Mitchell S.M., Sekikubo M., Byamugisha J.K., Kim J.J., Bryan S., Ogilvie G.S. (2017). Cost-effectiveness of cervical cancer screening methods in low- and middle-income countries: A systematic review. Int. J. Cancer.

[64] Sankaranarayanan R., Shyamalakumary B., Wesley R., Sreedevi Amma N., Parkin D.M., Krishnan Nair M. (1999). Visual inspection with acetic acid in the early detection of cervical cancer and precursors. Int. J. Cancer.

[65] Arbyn M., Buntinx F., Ranst M.V., Paraskevaidis E., Martin-Hirsch P., Dillner J. (2004). Virologic Versus Cytologic Triage of Women with Equivocal Pap Smears: A Meta-analysis of the Accuracy To Detect High-Grade Intraepithelial Neoplasia. JNCI J. Natl. Cancer Inst..

